# A Derivative of Plumbagin Targets the JAK2/STAT3 Pathway to Inhibit the Progression of Oral Squamous Cell Carcinoma

**DOI:** 10.3390/molecules31142419

**Published:** 2026-07-09

**Authors:** Xiyang Sun, Yiming He, Ting Xiao, Yuanmin Dong, Jiao Tian, Kaihua Wang, Henan Ma, Ziwen Wang, Honggang Zhou, Qingmin Wang, Cheng Yang

**Affiliations:** 1 State Key Laboratory of Medicinal Biology, College of Pharmacy, Nankai University, Tianjin 300353, China; 2120231650@mail.nankai.edu.cn (X.S.); h18102427783@163.com (Y.H.); 1120210467@mail.nankai.edu.cn (J.T.); kaikaiw71@gmail.com (K.W.); 2120211022@mail.nankai.edu.cn (H.M.); honggang.zhou@nankai.edu.cn (H.Z.); 2 Tianjin Key Laboratory of Molecular Drug Research, College of Chemistry, Nankai University, Tianjin 300353, China; 3 State Key Laboratory of Separation Membranes and Membrane Processes, School of Pharmaceutical Sciences, Tiangong University, Tianjin 300387, China; dongyuanmin@tiangong.edu.cn; 4 Tianjin Key Laboratory of Green Chemical Technology and Process Engineering, School of Pharmaceutical Sciences, Tiangong University, Tianjin 300387, China; 5 Tianjin Key Laboratory of Structure and Performance for Functional Molecules, College of Chemistry, Tianjin Normal University, Tianjin 300387, China; hxxywzw@tjnu.edu.cn; 6 Tianjin Key Laboratory of Molecular Drug Research, Tianjin International Joint Academy of Biomedicine, Tianjin 300457, China

**Keywords:** STAT3 inhibitor, plumbagin derivative, OSCC, ferroptosis

## Abstract

Signal transducer and activator of transcription 3 (STAT3) drives multiple hallmarks of tumorigenesis, making it a validated therapeutic target for diverse cancers. The 1,4-naphthoquinones, such as plumbagin (PL), exhibit anticancer activity via inhibiting STAT3 phosphorylation and dimerization, but their severe cytotoxicity precludes clinical translation. Here, we screened 23 PL derivatives for STAT3 inhibitory activity and normal cell cytotoxicity, identifying III-1a as the optimal candidate with superior STAT3 inhibition and reduced toxicity compared to PL. Molecular docking and cellular thermal shift assay (CTESA) revealed that III-1a binds to the L244 residue within the coiled-coil domain (CCD) of STAT3. In vitro, III-1a dose-dependently suppressed JAK/STAT3 signaling in oral squamous cell carcinoma (OSCC), particularly STAT3 Ser727 phosphorylation. It inhibited STAT3-mediated epithelial—mesenchymal transition (EMT) and induced ferroptosis, thereby attenuating OSCC proliferation, invasion and migration. STAT3 knockdown abrogated its anti-tumor effects, and in vivo studies further confirmed its efficacy against OSCC growth. Collectively, this study identifies a novel PL-derived STAT3 inhibitor targeting STAT3 CCD to regulate EMT and ferroptosis, providing a promising therapeutic candidate for OSCC.

## 1. Introduction

Oral squamous cell carcinoma (OSCC) is a common malignant tumor of the head and neck, accounting for 90% of all malignant tumors occurring in the oral cavity. According to global cancer statistics, there were 377,713 new cases of OSCC worldwide in 2020 [1], with the majority of cases occurring in the Asian region. The primary treatment for OSCC is surgical resection, and adjuvant radiotherapy, chemotherapy, targeted therapy, or immunotherapy may be administered when necessary [2]. However, OSCC often causes disfigurement and functional impairments in patients, including difficulties with swallowing, speech, and taste perception, which severely compromise patients’ quality of life. It is particularly important to explore the therapeutic targets and drugs of OSCC.

The IL6/JAK/STAT3 signaling pathway plays a major role in the progression of cancer, participating in multiple biological processes including cell proliferation, survival, angiogenesis, invasion, metastasis, immune evasion, and chemotherapy resistance [3,4,5,6]. During tumor development, elevated levels of IL6 in the tumor microenvironment lead to sustained overactivation of the JAK/STAT3 signaling pathway, which is often associated with poor prognosis in patients [7]. Activation of STAT3 in tumor cells is not merely regulated by cytokines and their receptors, but also by receptor tyrosine kinases and some non-receptor tyrosine kinases [8,9]. As a transcriptional regulatory factor, phosphorylated and activated STAT3 dimerizes and translocates into the nucleus to regulate the transcription of numerous target genes, thereby participating in tumor initiation and malignant progression [10,11].

A large number of studies have reported that STAT3 inhibitors or knockout systems can effectively block the activation of STAT3 and inhibit the progression of cancer [12]. The currently accepted anti-tumor mechanism of STAT3 inhibitors involves directly targeting the STAT3 protein and blocking the upstream signaling pathways of STAT3. Inhibitors that block the upstream signaling of STAT3 mainly focus on inhibiting the interacting proteins of STAT3, such as JAK, RTK, Src, and the activating factor IL6 [13,14,15]. Direct inhibitors of STAT3 typically act on the structural domains of STAT3, such as Src homology 2 domain (SH2), DNA-binding domain (DBD), and Transactivation domain (TAD), to block the phosphorylation, dimerization, nuclear translocation, and DNA binding of STAT3 [16,17]. The progress of STAT3 inhibitors in anti-tumor drug development suggests that STAT3 can be an attractive target for cancer treatment.

Naphthoquinone is a well-recognized privileged pharmacophore in anticancer drug discovery, characterized by its planar aromatic structure, redox-active quinone moiety, and ability to form multiple non-covalent interactions (hydrogen bonds, π-π stacking, and salt bridges) with target proteins. Extensive preclinical and clinical studies have confirmed that naphthoquinone derivatives potently inhibit STAT3 signaling transduction by targeting the SH2 domain or suppressing STAT3 phosphorylation [18,19]. For instance, Yu et al. reported a landmark naphthoquinone-based STAT3 inhibitor, compound 9 (5,8-dioxo-6-((2-(piperazin-1-yl)phenyl)-amino)-5,8-dihydronaphthalene-1-sulfonamide), which was identified via advanced multiple ligand simultaneous docking (AMLSD). This compound exhibited potent STAT3 inhibitory activity and anti-breast cancer efficacy, representing a major breakthrough in the development of naphthoquinone-derived STAT3 inhibitors [20]. Numerous studies have also documented natural naphthoquinones with STAT3 inhibitory activity and anti-tumor effects, including napabucasin, cryptotanshinone, and plumbagin [21]. Recently, plumbagin (PL) has become a hot topic in anti-tumor research. It has been reported that PL can participate in tumor cell progression by regulating the expression of STAT3, NF-κB and AKT [22,23]. Several studies have reported that PL inhibits pancreatic cancer, breast cancer, esophageal squamous cell carcinoma, melanoma, prostate cancer and gastric cancer by directly or indirectly acting on STAT3, playing a unique role in cancer treatment [24,25]. It regulates cancer cell proliferation, apoptosis, autophagy, angiogenesis, invasion and metastasis [26]. Although PL is a potential anticancer drug, its high cytotoxicity and poor water solubility limit its clinical application [27]. Therefore, it is hoped that more valuable anti-tumor drugs can be developed through structural optimization of PLs to improve their physical and chemical properties and retain or enhance their anti-tumor activities. In this study, we designed and synthesized a series of derivatives of PL. Among them, we determined compound III-1a that exhibited lower toxicity and better STAT3 inhibitory activity than PL and further elucidated its anti-tumor effects and mechanisms in OSCC.

## 2. Results

### 2.1. III-1a Targeted STAT3 Inhibits the Activation of JAK/STAT3 Signal Pathway

**Figure 1:**
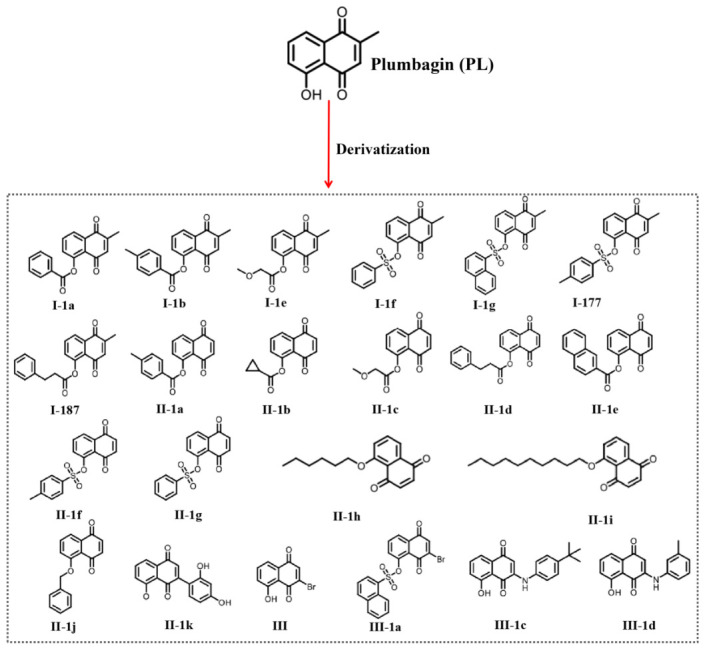
Structure of PL and 23 kinds of derivatives.

**Figure 2:**
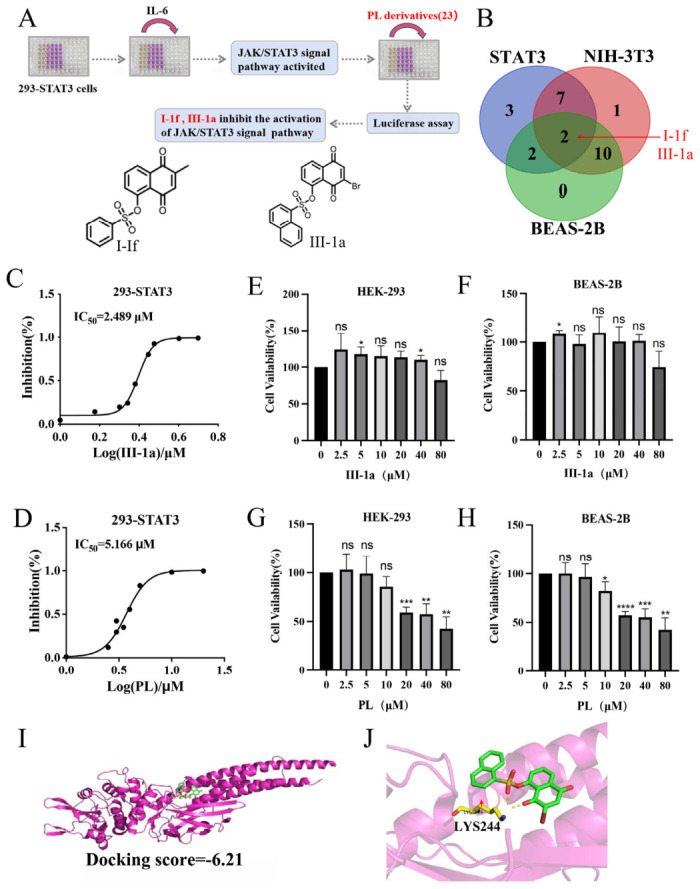
The STAT3 inhibitory activity of PL derivative III-1a is better than that of PL. (**A**) Schematic diagram of STAT3 signaling pathway inhibitor activity screening. (**B**) Venn analysis of high STAT3 inhibitory activity and low cytotoxicity of NIH3T3 and BEAS-2B cells. (**C**) The IC_50_ of STAT3 inhibitory activity of III-1a. (**D**) The IC_50_ of STAT3 inhibitory activity of PL. (**E**) The toxicity of III-1a to HEK-293 cells within 80 μM. (**F**) The toxicity of III-1a to BEAS-2B cells within 80 μM. (**G**) The toxicity of PL to HEK-293 cells within 80 μM. (**H**) The toxicity of PL to BEAS-2B cells within 80 μM (Data represent mean ± SD of three separate experiments. * *p* < 0.05, ** *p* < 0.01, *** *p* < 0.001, **** *p* < 0.0001.). (**I**,**J**) Molecular docking analysis of III-1a and STAT3.

We previously synthesized 23 PL derivatives as described in our study [28] and shown in Figure 1. Using a luciferase assay, we evaluated the inhibitory effects of PL and its derivatives on JAK/STAT3 pathway activation and the cytotoxicity to NIH-3T3 and BEAS-2B cells (Figure 2A and Figure S1A); notably, the inhibition rates of 12 PL derivatives exceeded 80% (Table 1). Our results identified two compounds, I-1f and III-1a, as effective STAT3 inhibitors with minimal toxicity to normal cells at a 20 μM concentration (Figure 2B). However, I-1f showed a paradoxical activation of the STAT3 pathway at higher concentrations (Figure S1B). The IC_50_ values for III-1a and PL against the STAT3 pathway were determined to be 2.489 μM and 5.166 μM, respectively (Figure 2C,D). Further cytotoxicity assays on HEK-293 and BEAS-2B cells revealed that PL exhibited greater toxicity compared to III-1a (Figure 2E–H). Additionally, the results of molecular docking suggest that compound III-1a forms a relatively stable hydrogen bond with the Lys244 residue within the coiled-coil domain of the STAT3 protein, with a docking score of −6.21, indicating a favorable binding affinity between the compound and STAT3 (Figure 2I,J). We also performed molecular docking to evaluate the binding affinity between candidate molecules with potent STAT3 inhibitory activity and the STAT3 protein. The results demonstrated that the docking scores of all 11 candidate molecules and PL were lower in absolute value than that of III-1a (Figure S2). Additionally, we performed MM-GBSA calculations for III-1a and STAT3. AutoDock Vina generated 20 docking poses for compound III-1a in the binding pocket proximal to Lys244 of STAT3. The docking scores of the top 10 poses range from −7.220 to −6.993 kcal/mol. All 10 top-ranked poses maintain contact with Lys244 within a 4 Å cutoff (Table S1), among which poses 2, 4, 5, 7, and 9 display potential hydrogen-bonding interactions with Lys244. Following MM-GBSA rescoring of the top five poses, pose 2 yields the most favorable ΔG_bind (−2.2531 kcal/mol), while preserving a good docking score (−7.203 kcal/mol) and a putative NZ-O contact with Lys244 at approximately 3.34 Å (Table S2, Figure S3). Accordingly, pose 2 was selected as the final binding conformation of the III-1a–STAT3 complex.

**Figure 3:**
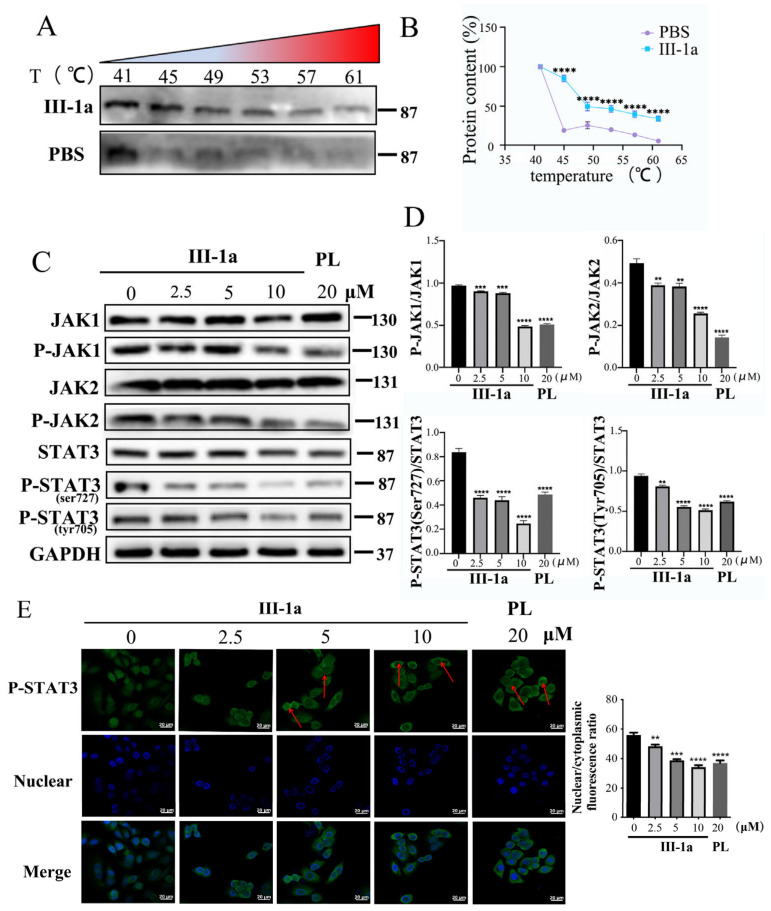
III-1a inhibits the activation of JAK/STAT3 signal pathway. (**A**,**B**) CETSA analysis of the binding strength of III-1a to STAT3 in CAL27 cells at different temperatures with PBS as a negative control. (**C**,**D**) Western blot analysis: The expression of JAK1, P-JAK1, JAK2, P-JAK2, STAT3, P-STAT3 (ser727), p-STAT3 (tyr705) and their gray level in CAL27 cells treated with different concentrations of III-1a and 20 μM PL. (**E**) Immunofluorescence images of p-STAT3 in CAL27 cells (40×), the red arrow indicates the position of the cell nucleus. After treatment with III-1a and PL, the green fluorescence in the cell nucleus was weakened. Data represent mean ± SD of three separate experiments. ** *p* < 0.01, *** *p* < 0.001, **** *p* < 0.0001.

To assess the anticancer efficacy of III-1a, we examined its impact on the cell viability of 11 types of cancer cells. We found that III-1a exhibited the best anti-tumor activity against oral squamous cell carcinoma (CAL27 cells), with an IC_50_ value of 30 ± 2.13 μM (Table 2, Figure S1C–M). Additionally, to further evaluate the inhibitory effect of III-1a on STAT3, we performed cellular thermal shift assays. The results indicated that III-1a binds to STAT3, stabilizing it within a temperature range of 41–61 °C and preventing its degradation (Figure 3A,B). Small molecules immobilized on CNBr-activated Sepharose 4B beads enable the capture of their cognate interacting proteins from cellular lysates. To further confirm the direct interaction between compound III-1a and STAT3, we incubated cell lysates with CNBr-activated Sepharose 4B beads conjugated with III-1a (designated CNBr-III-1a) and control beads treated with DMSO (designated CNBr-DMSO), respectively, to pull down small molecule-binding proteins. The results demonstrated that STAT3 protein was efficiently enriched in the CNBr-III-1a group, whereas no detectable STAT3 was recovered from the CNBr-DMSO control group, supporting a direct physical interaction between compound III-1a and STAT3 (Figure S4A). Western blot analysis revealed that III-1a more effectively inhibited the phosphorylation of JAK1, JAK2, and STAT3 (ser727 and tyr705) compared to PL (Figure 3C,D). Immunofluorescence studies of III-1a and PL-treated CAL27 cells showed that both compounds could inhibit the nuclear translocation of STAT3 (Figure 3E). Our findings preliminarily suggest that III-1a targets STAT3 to more effectively inhibit its phosphorylation than PL.

### 2.2. III-1a Inhibits the Invasion, Migration and Proliferation of Oral Squamous Cell Carcinoma Cells

**Figure 4:**
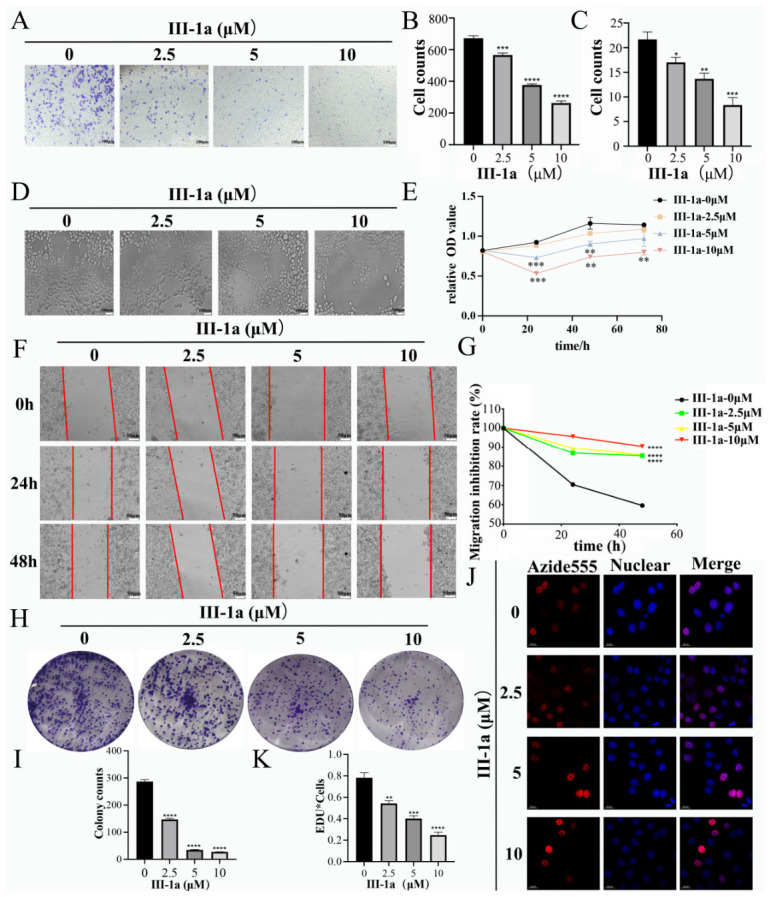
III-1a inhibits the proliferation, invasion, vasculogenic mimicry (VM) formation, and migration of oral squamous cell carcinoma cells. (**A**,**B**) III-1a (0 μM, 2.5 μM, 5 μM and 10 μM) attenuated CAL27 cells invasion after 24 h; the number of invasive cells was calculated by quantitative analysis. (**C**,**D**) The ability of III-1a to inhibit the formation of VM was evaluated by tube formation in CAL27 cells. (**E**) The survival rate of CAL27 cells was detected at four time points of 0, 24, 48, 72 h by MTT assay and cell proliferation curves were plotted. (**F**,**G**) The inhibition of III-1a on migration of CAL27 cells within 48 h by wound healing assay. (**H**,**I**) III-1a inhibits the colony formation of CAL27 cells. (**J**,**K**) The inhibitory effect of III-1a on the proliferation of CAL27 cells was detected by EDU staining. Data represent mean ± SD of three separate experiments. * *p* < 0.05, ** *p* < 0.01, *** *p* < 0.001, **** *p* < 0.0001.

We further evaluated the inhibitory effects of III-1a on oral squamous cell carcinoma using CAL27 cells. Transwell, 3D culture, wound healing and colony formation assays were used to assess the impact of III-1a on CAL27 cell invasion, tube formation, migration, and colony formation abilities; the results show that III-1a significantly inhibits invasion (Figure 4A,B), vasculogenesis mimicry formation (Figure 4C,D), migration (Figure 4F,G), and colony formation abilities (Figure 4H,I) of CAL27 cells in a dose-dependent manner within 10 µM. Proliferation curves and EDU staining assays were used to evaluate cell proliferation and DNA replication of CAL27 cells; the proliferation (Figure 4E) and DNA replication (Figure 4J,K) of CAL27 cells were obviously reduced after treatment with III-1a.

### 2.3. III-1a Inhibits EMT and Induces Ferroptosis in Oral Squamous Cell Carcinoma Cells

**Figure 5:**
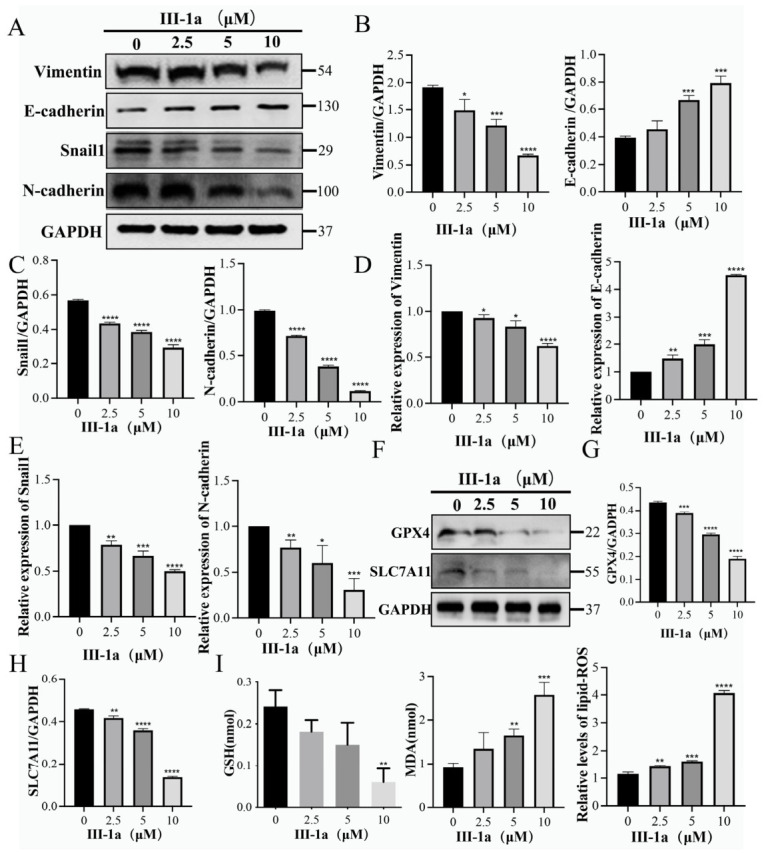
III-1a inhibits EMT and induces ferroptosis in oral squamous cell carcinoma cells. (**A**–**C**) Western blot and statistical analysis of the expression of Vimentin, E-cadherin, Snail 1, and N-cadherin in CAL27 cells treated with III-1a. (**D**,**E**) qPCR and statistical analysis of the mRNA levels of Vimentin, E-cadherin, Snail 1, and N-cadherin in CAL27 cells treated with III-1a. (**F**–**H**) Western blot and statistical analysis of the expression of SLC7A11 and GPX4 in CAL27 cells treated with III-1a. (**I**) Detection and statistical analysis of intracellular GSH, MDA, and lip-ROS levels in CAL27 cells treated with III-1a. Data are presented as means ± SD, *n* = 3. * *p* < 0.05, ** *p* < 0.01, *** *p* < 0.001, **** *p* < 0.0001.

Tumor epithelial–mesenchymal transition (EMT) is a key mechanism that promotes tumor proliferation and metastasis. In our research, we evaluated whether III-1a inhibits the EMT process in OSCC by targeting STAT3. Western blot and qPCR were used to assess the differences in protein and mRNA levels of EMT markers in CAL27 cells treated with III-1a. Our findings showed that III-1a dose-dependently increases the expression of the epithelial marker E-cadherin and decreases the expression of mesenchymal markers N-cadherin, Snail, and Vimentin, suggesting that III-1a may inhibit the EMT process in OSCC (Figure 5A–E). Additionally, it was reported that STAT3 participates in regulating ferroptosis, which is a novel and iron-dependent cell death, so we further examined whether III-1a could inhibit OSCC cell proliferation by inducing ferroptosis. We analyzed the expression of key proteins regulating lipid peroxidation, Solute Carrier Family 7 Member 11 (SLC7A11, xCT) and Glutathione Peroxidase 4 (GPX4), in CAL27 cells treated with various concentrations of III-1a. We observed a significant reduction in the protein expression of SLC7A11 and GPX4 (Figure 5F–H). Additionally, we first assessed the effect of III-1a on the viability of CAL27 cells. The results showed that within the concentration range of 10μM, the ratio of viable CAL27 cells to total cells did not exhibit a significant change (Figure S4B); subsequently, we treated CAL27 cells with a combination of Erastin and RSL3 at different concentrations of III-1a, and cell viability was assessed after 24 h. Our results showed that both Erastin and RSL3 significantly inhibited CAL27 cell viability when treated separately, while the combination of different concentrations of III-1a with Erastin and RSL3 further enhanced the inhibitory effect on CAL27 cells. Importantly, this inhibition was partially reversed when the ferroptosis inhibitor Ferrostain-1 was added; co-treatment with Ferrostatin-1 increased cell viability to 1.34-fold of that observed with III-1a monotherapy for the 2.5 μM and 5 μM III-1a treatment groups, respectively, further indicating that III-1a may enhance the effects of Erastin and RSL3 by inducing ferroptosis (Figure S4C,D). Moreover, after treatment with III-1a, we measured changes in the levels of MDA, GSH, and lip-ROS in the cells, noting an increase in lip-ROS and MDA levels and a decrease in GSH levels (Figure 5I). These preliminary changes in biomarkers and biochemical factors suggest that III-1a may not only inhibit cellular EMT but also induce ferroptosis.

### 2.4. Knocking Down STAT3 Weakens the Effect of III-1a on EMT Process and Ferroptosis

**Figure 6:**
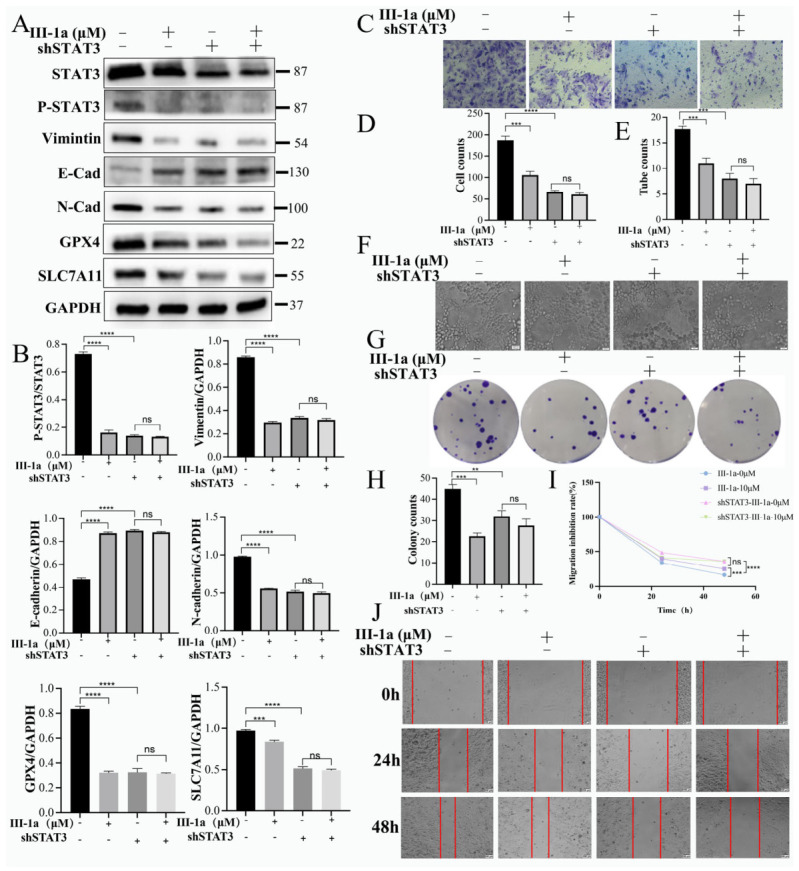
III-1a suppresses the EMT and promotes ferroptosis of CAL27 cells by targeting STAT3. (**A**,**B**) The expression of STAT3, p-STAT3, EMT marker and ferroptosis marker in STAT3 knockdown CAL27 cells was detected by Western blot. (**C**–**J**) Knockdown of STAT3 expression affected the inhibitory effect of III-1a (10 μM) on CAL27 cell invasion (**C**,**D**), VM formation (**E**,**F**), colony formation (**G**,**H**) and migration (**I**,**J**). Data are presented as the means ± SD, *n* = 3. ** *p* < 0.01, *** *p* < 0.001, **** *p* < 0.0001.

In previous research, we revealed that III-1a targets STAT3 and inhibits the proliferation and metastasis of oral squamous cell carcinoma cells. Additionally, we have preliminarily discovered that treatment with III-1a leads to changes in markers related to EMT and ferroptosis in the cells. In order to further determine the effect of III-1a on STAT3-mediated EMT and ferroptosis, we knocked down the expression of STAT3 in CAL27 cells. The levels of EMT, ferroptosis markers and phosphorylated STAT3 were detected by Western blot. The results showed that the expression of Vimentin, N-cadherin, GPX4, SLC7A11 and the phosphorylation of STAT3 were significantly down-regulated and the expression of E-cadherin was up-regulated by treated with III-1a alone or knocking down the expression of STAT3. Compared with the STAT3 knockdown group, the treatment of knocking down STAT3 and adding III-1a did not noticeably decrease the expression of Vimentin, N-cadherin, GPX4 and SLC7A11 and phosphorylation of STAT3, and did not significantly up-regulate the expression of E-cadherin (Figure 6A,B). Then we evaluated the effects of knockdown STAT3 on the migration, invasion, VM formation, and colony formation of CAL27 cells. As before, treatment with III-1a or knockdown STAT3 alone could significantly inhibit the ability of cell migration, invasion, VM formation, and colony formation, but there was no significant difference in the ability of migration, invasion, VM formation, and colony formation in STAT3 knockdown cells treated with 10 μM III-1a (Figure 6C–J). These results suggest that knocking down the expression of STAT3 in CAL27 cells can weaken the effects of III-1a on cell migration, invasion, VM formation, and colony formation, as well as EMT and ferroptosis.

### 2.5. III-1a Inhibits the Growth of Oral Squamous Cell Carcinoma In Vivo

**Figure 7:**
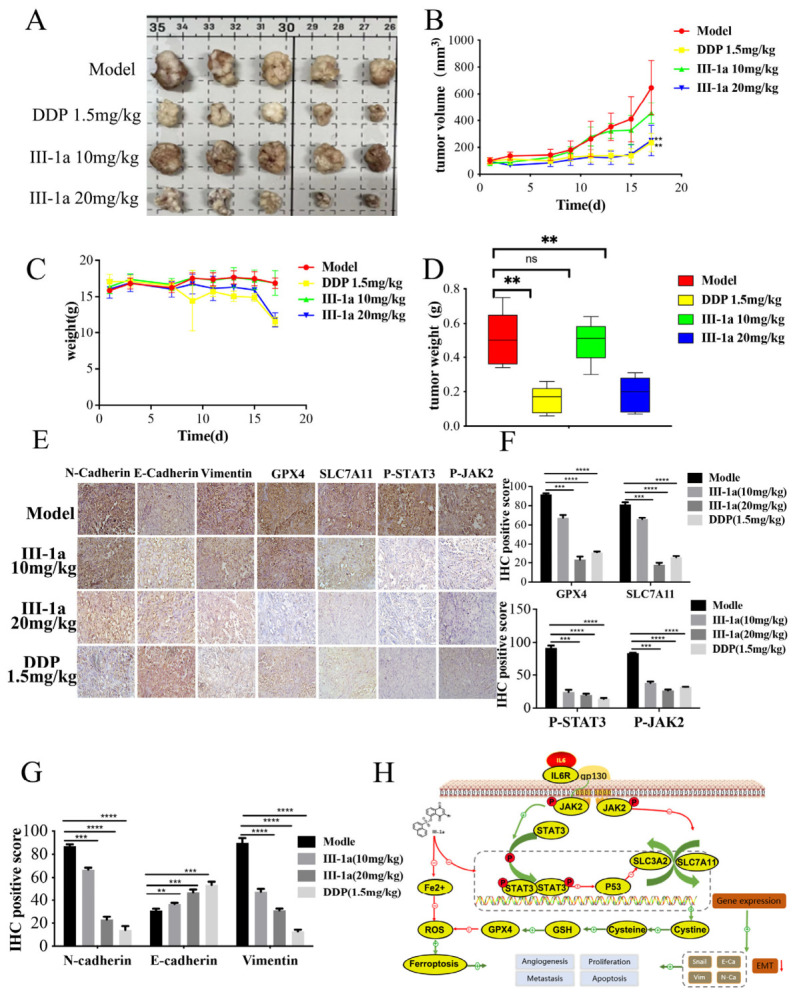
III-1a suppressed the tumor growth of oral squamous cell carcinoma. (**A**) Picture of a nude mouse tumor with CAL27 cells. (**B**) Tumor volume in mice during the 18-day administration cycle. (**C**) The body weight of mice during the 18-day administration cycle. (**D**) Tumor weight of mice on the 18th day after administration. (**E**–**G**) The effect of III-1a on the content of related marker proteins in tumor tissues was analyzed by IHC assay. (**H**) Mechanism for the anti-tumor effect of III-1a, The red arrow indicates that the expression of EMT-related proteins is down-regulated and the EMT process is inhibited. Data are presented as the means ± SD, *n* = 5. ** *p* < 0.01, *** *p* < 0.001, **** *p* < 0.0001.

In order to confirm whether III-1a can inhibit tumor proliferation in vivo, we established a CAL27 transplanted tumor model in Balb/c nude mice. The mice were randomly divided into four groups: model group (saline), cisplatin group (1.5 mg/kg/d), III-1a low-dose group (10 mg/kg/d) and III-1a high-dose group (20 mg/kg/d). The data of tumor volume and tumor weight showed that III-1a could inhibit the growth of CAL27 tumor in a dose-dependent manner. We also evaluated the anti-tumor activity of PL in vivo. The results showed that the tumor inhibition rate of III-1a 20 mg/kg group was 63.5%, similar to 69.8% of cisplatin group, while the tumor inhibition rate of PL 20 mg/kg was only 43.2% (Figure 7A,B,D and Figure S5). However, the high dose group of III-1a and cisplatin could reduce the body weight of mice to some extent, especially on the 18th day of treatment (Figure 7C). Next, we detected the expression of N-cadherin, E-cadherin, Vimentin, GPX4, SLC7A11 and phosphorylated STAT3 and JAK2 in tumor tissues by IHC. The results showed that III-1a could down-regulate the expression of N-cadherin, Vimentin, GPX4, SLC7A11, P-STAT3 and P-JAK2 in a dose-dependent manner, while it could up-regulate the expression of E-cadherin. Cisplatin also had a significant effect on the expression of EMT and ferroptosis marker proteins and the phosphorylation of JAK2/STAT3 pathway in CAL27 tumors (Figure 7E,F). To sum up, we concluded that III-1a can inhibit the growth of oral squamous cell carcinoma by affecting STAT3-mediated EMT and ferroptosis. Finally, we described the molecular mechanism and signal pathway of the anti-oral squamous cell carcinoma effect of III-1a (Figure 7H).

## 3. Discussion

Overactivation of STAT3 plays a role in the progression and metastasis of OSCC and is closely related to the clinical stage and poor prognosis of patients [29,30]. Targeting STAT3 protein is a potential and promising strategy for the treatment of OSCC. In this study, through the screening of STAT3 pathway activation inhibitors, we found for the first time that III-1a, a structural analogue of PL, can inhibit the migration and invasion of OSCC cells by inhibiting the activation of JAK/STAT3 signal pathway and has a good therapeutic effect on oral squamous cell carcinoma in vivo.

In recent years, the development of inhibitors of JAK/STAT3 signaling pathway has become a hotspot in the research of antineoplastic drugs. At present, many types of JAK/STAT3 pathway inhibitors have carried out preclinical and clinical studies on anti-tumor effects, including peptides, oligonucleotides, antibodies and small molecules [31]. Non-peptide small molecules have many advantages, which makes scientists devote themselves to the development of STAT3 small molecule inhibitors. Although there are no STAT3 inhibitors on the market, some of them have successfully entered clinical trials [32]. Quinones have been reported to be a class of potential STAT3 inhibitors, such as benzoquinone BPMP, naphthoquinone atovaquone and plumbagin and their derivatives have good STAT3 inhibitory activity and anti-tumor activity [21]. 1-4-naphthoquinone is a kind of important small molecular compounds for the design of anticancer drugs. Bhasin and his colleagues studied the structure–activity relationship of 1-naphthoquinone derivatives and found that the single substitution at C2 or C3 position could increase the anti-tumor proliferative activity of the compound, while the disubstituted compound could decrease the activity. The existence of hydroxyl group at position C5 has little effect on anti-proliferation activity, but the substitution of sulfonyl group at position C5 is the key to bind to the SH2 domain of STAT3 protein and prevent its dimerization [19]. In our study, we found that C5 biphenyl sulfonate substitution and C3 bromination (III-1a) can maintain the STAT3 activity and reduce the cytotoxicity of the compounds. In addition, compared with III-1a, the lack of C5 biphenylsulfonate substitution (III) greatly reduces the STAT3 activity, and the methyl substitution at the C2 position (III-1g) instead of the bromine substitution at the C3 position also reduces the STAT3 activity. Li and colleagues [33] found that the C5 position of plumbagin is more important to the activity, and the hydroxyl at position 5 may interact with STAT3 in the form of hydrogen bond, which is beneficial to the binding with STAT3. The introduction of additional groups or suitable linkers in the aromatic ring can also promote the activity. In this study, we found that III-1a had better STAT3 inhibitory activity and weaker cytotoxicity compared with PL. The results of our molecular docking showed that the binding site between III-1a and STAT3 was Lys244 amino acid, not the TAD domain of PL interaction. It is possible that the hydroxyl substituent at position 5 changed the way the compound binds to STAT3. The binding energy of molecular docking is −6.21, which still shows that III-1a has a certain binding effect with STAT3. However, molecular docking scores merely reflect preliminary binding propensity and predicted binding sites; accurate binding affinity requires further validation through more precise computational approaches and biophysical experiments, including MM-GBSA calculations. Nonetheless, in the present study, the direct interaction between III-1a and STAT3 was verified via cellular thermal shift assay (CETSA) and CNBr-activated Sepharose 4B pull-down assay. We also confirmed that III-1a suppresses the phosphorylation of JAK/STAT3 pathway and inhibits the nuclear translocation of p-STAT3.

Vasculogenic mimicry (VM) describes a process whereby highly invasive tumor cells undergo phenotypic reprogramming and extracellular matrix remodeling to spontaneously form blood-perfused, vessel-like tubular structures that supply nutrients and oxygen to tumor cells. Epithelial–mesenchymal transition (EMT) acts as a core driver of VM formation [34]. In addition, accumulated evidence has demonstrated that both EMT and ferroptosis participate in tumorigenesis, invasion, metastasis, and chemoresistance, and STAT3 modulates EMT and ferroptosis processes across multiple tumor types [35,36]. The STAT3 inhibitor III-1a identified in this study suppresses EMT, proliferation, invasion, and vasculogenic mimicry formation, while promoting ferroptosis, in oral squamous cell carcinoma cells in a dose-dependent manner. Knockdown of STAT3 attenuates the regulatory effects of III-1a on EMT, proliferation, invasion, vasculogenic mimicry formation, and ferroptosis. However, despite the confirmed favorable anti-tumor efficacy and STAT3 inhibitory activity of compound III-1a both in vitro and in vivo, we observed body weight loss in mice from the high-dose III-1a group at the end of the administration period in the in vivo efficacy study. The in vivo safety profile of III-1a has yet to be fully characterized. In subsequent work, systematic toxicity evaluations—including histopathological examination, serum biochemical analysis, and hematological testing—will be conducted for both acute and chronic toxicity, to clarify the toxicological properties and safe therapeutic window of III-1a.

In short, our study reported for the first time that PL derivatives III-1a inhibit the activation of JAK/STAT3 signal pathway by targeting STAT3 to inhibit EMT and promote ferroptosis in oral squamous cell carcinoma. III-1a is expected to be developed as a potential therapeutic drug for oral squamous cell carcinoma.

## 4. Materials and Methods

### 4.1. Compounds

The compounds tested in this study were synthesized and provided by Prof. Wang’s laboratory, School of Chemistry, Nankai University according to previous reports [28].

### 4.2. Cell Lines and Cell Culture

All cell lines used in this study were purchased from Shanghai Fuheng Biotechnology Co., Ltd. (Shanghai, China). The FADU, CAL27, SKOV3, KYSE, MCF-7, 5637, PANC-1, NOZ, KB, HEK-293, NIH-3T3, and BEAS-2B cell lines were maintained in Dulbecco’s Modified Eagle Medium (DMEM; Solarbio, Beijing, China), while A549 and H460 cells were cultured in RPMI-1640 medium. All media were supplemented with 10% (*v*/*v*) fetal bovine serum (FBS; Procell, South America origin). The cells were incubated at 37 °C in a humidified atmosphere containing 5% CO_2_ and 95% air [37].

### 4.3. Animal and Immunohistochemical Staining

The BABL/c nude mice (5–6 wk) were purchased from Charles River Laboratory in Beijing, China. The mice were separately fed under SPF barrier. CAL27 cells (1 × 10^7^ cells per mice) were injected subcutaneously into the left foreleg of nude mice to establish a tumor model. The mice were randomly divided into four groups with 5 mice per group. Normal saline (0.9% NaCl) group or model group, Cisplatin (DDP) group (1.5 mg/kg/2 days), III-1a low-dose group (10 mg/kg/d), III-1a high-dose group (20 mg/kg/d). On the 7th day of modeling, mice in groups III-1a were administrated by intragastric administration every day, and mice in cisplatin group were administrated by intraperitoneal injection every other day. The tumor volume (V = L × W2/2) and body weight of mice were recorded every two days within 18 days of administration. Due to its ability to suppress the respiratory center, an overdose of pentobarbital sodium can lead to asphyxiation and accompanying cardiac arrest, causing animals to peacefully pass away without experiencing significant discomfort. Therefore, 18 days after drug administration, mice were euthanized by intraperitoneal injection of 150 mg/kg pentobarbital sodium, inducing euthanasia through overdose-induced deep anesthesia. After euthanasia, the xenograft tumors of each mouse were weighed and analyzed. Each tumor was immobilized with 10% formalin. Then, the tissue sample is embedded and sliced.

After the slices were dewaxed with xylene and hydrated with fractional alcohol, they were soaked in citric acid antigen recovery solution and heated in microwave oven. According to the immunohistochemical kit (Maxim Biotech, Shanghai, China), the sections were combined with primary antibody (E-cadherin, N-cadherin (22018-1-AP), Vimentin (cat.AF0071), p-STAT3 (cat.AF3295), and p-JAK2 (cat.AF3024), GPX4 (cat.DF6701) and Slc7a11 (cat.DF12509) were incubated at 4 °C overnight and incubated with biotin-labeled secondary antibodies at room temperature for 30 min. Color reaction was performed by enzyme-linked polymer detection system and hematoxylin reverse staining. The staining of sections of each group was compared.

### 4.4. Cell Viability Assay

CAL27 cells were seeded in 96-well plates at a density of 5 × 10^4^ cells/well and allowed to adhere for 24 h. Cells were then treated with varying concentrations of III-1a for 48 h. Following treatment, 20 μL of MTT solution (5 mg/mL) was added to each well, and the plates were incubated at 37 °C for 4 h. The formazan crystals were solubilized with DMSO, and absorbance was measured at 570 nm using a microplate reader (Multiskan FC, Thermo Scientific, Waltham, MA, USA). Data were analyzed using GraphPad Prism 9.0, and all experiments were performed in at least triplicate.

### 4.5. Luciferase Assay

First, the STAT3 gene plasmid with a luciferase tag was synthesized by GeneCopoeia (Guangzhou, China), and the plasmid was transfected into HKE-293 using LipofectamineTM 2000 (Thermo Fisher Scientific, cat.11668027, Waltham, MA, USA) according to the reagent instructions to construct a stable cell line named (293-STAT3). Before the luciferase assay, 293-STAT3 was seeded into a 96-well plate at 5000 cells/well, and the cells were cultured for 48 h with different compounds and IL-6 (70 ng/mL). Then, the luciferase fluorescence assay was performed using the assay kit (Beyotime, cat. RG126M, Shanghai, China) and the kit instructions were followed. The culture medium was discarded; the cells were washed thrice with PBS and then added with 50 μL of cell lysis buffer. The plate was placed on an oscillator at 120 r/min for 30 min, 35 μL/well of cell lysate was transferred to the white plate, 40 μL of diluted luciferase detection substrate was added to each well, and the fluorescence value was detected using a luciferase fluorescence detector (Promega, Madison, WI, USA).

### 4.6. Wound Healing Assay

CAL27 cells were trypsinized, centrifuged, and seeded into 24-well plates. Upon reaching 100% fusion, a linear scratch was introduced in the monolayer using a sterile pipette tip. The cells were then incubated in serum-free medium supplemented with III-1a (0, 2.5, 5, or 10 μM). Wound closure was monitored at 0, 24, and 48 h, with images captured using an inverted microscope (Nikon, Yokohama, Japan). Migration distances were quantified using ImageJ 1.54g software (NIH, Stapleton, NY, USA). All experiments were performed in triplicate.

### 4.7. Cellular Thermal Shift Assay (CETSA)

CAL27 cells were treated with PBS or III-1a (10 μM) for 4 h and then digested by trypsin and collected by 300× *g* centrifugation. Cell suspension was divided into 100 μL tubes after washing with PBS or III-1a (10 μM) and heated at different temperatures (41, 45, 49, 53, 57, 61 °C) for 5 min. Then cell suspension was freeze–thawed 3–4 times repeatedly between room temperature and liquid nitrogen, and finally centrifuged at 20,000× *g* at 4 °C for 20 min. The supernatant was collected for Western blot analysis.

### 4.8. RNA Extraction and Real-Time Fluorescence Quantitative PCR

Total RNA was extracted from CAL27 cells with Trizol (Solarbio) reagent, and its concentration was determined by thermo-nanodrop one (Thermo, Wilmington, DE, USA). cDNA was synthesized by reverse transcription using fastking gDNA dispelling RT Super Mix (TIANGEN, Beijing, China). Then, qPCR SYBR Green Master Mix (Yeason, Shanghai, China) was used for quantitative PCR.

### 4.9. Western Blot (WB) Analysis

Cells (CAL27) were treated with III-1a (0 μM, 2.5 μM, 5 μM, and 10 μM) and PL (20 μM) for 24 h and lysed with RIPA buffer, and the total protein concentration of CAL27 cells was determined by BCA kit (Solarbio, Beijing, China). Equivalent protein samples were separated by 8–15% gels and transferred to PVDF membranes (Merck, Darmstadt, Germany). After being enclosed in 5% buttermilk at room temperature for 1 h, the corresponding primary antibody (1:1000) was incubated at 4 °C overnight, and the secondary antibody (1:10,000) was incubated at room temperature for 2 h. Goat anti-Rabbit antibody was obtained from Cell Signaling Technology Inc. (Beverly, MA, USA). The immune response was detected by ECL reagent (Affinity). The gray scale of strip is quantitatively analyzed by ImageJ. The antibodies used include E-cadherin (cat.AF0131), N-cadherin (22018-1-AP), Vimentin (cat.AF0071), Snail1 (cat.AF6032), STAT3 (cat.AF0294), p-STAT3 (cat.AF3295), JAK2 (cat.AF0022), p-JAK2 (cat.AF3024), GAPDH (cat.AF7021), JAK1(cat.AF7765), P-JAK1 (cat.AF7265), P-STAT3 (Ser727) (cat.9134S), P-STAT3 (Tyr705) (cat.9145S), GPX4 (cat.DF6701), and SLC7A11 (cat.DF12509).

### 4.10. EDU (5-Bromo-2-deoxyuridine) Detection of Cell Proliferation

EdU (5-ethynyl-2′-deoxyuridine), a thymidine analog that incorporates into newly synthesized DNA during cell proliferation, was used to assess CAL27 cell proliferation. Following 24 h treatment with III-1a (0, 2.5, 5, or 10 μM), cells were incubated with EdU working solution (Solarbio, Beijing, China) according to the manufacturer’s protocol. Subsequently, cells were fixed and stained using Click reaction cocktail for 30 min under light-protected conditions. EdU-positive cells were visualized and quantified using a laser scanning confocal microscope (SP800).

### 4.11. Transwell Assay

Cell invasive capacity was evaluated using 24-well transwell chambers with 8.0 μm pore membranes. The membranes were pre-coated with Matrigel (diluted 1:3 in serum-free DMEM) and polymerized at 37 °C for 30 min. Following hydration with 0.1% FBS medium for 30 min, CAL27 cells (5 × 10^4^ cells/well) suspended in 0.1% FBS medium were seeded into the upper chamber. The lower chamber contained 500 μL of complete medium with 10% FBS as a chemoattractant. After 24 h incubation at 37 °C in 5% CO_2_, non-invaded cells on the upper membrane surface were removed using cotton swabs. Invaded cells were fixed with 4% paraformaldehyde for 15 min, stained with 0.1% crystal violet, and quantified under an inverted microscope (Nikon Eclipse Ti, Yokohama, Japan) in five random fields per well.

### 4.12. Tube Formation Assay

Tube formation was assessed using a Matrigel-based angiogenesis assay. Briefly, 50 μL of growth factor-reduced Matrigel (ABW^®^, Shanghai, China) was mixed with serum-free DMEM (1:1 ratio) and polymerized in 96-well plates at 37 °C for 20 min. CAL27 cells (1 × 10^4^ cells/well in 100 μL medium) were then seeded onto the polymerized Matrigel and cultured for 24 h at 37 °C in a 5% CO_2_ humidified atmosphere. Tubular structures were visualized under an inverted microscope (40× magnification), with five random fields captured per well. Tube formation was quantified by counting branch points and total tube length using ImageJ software (NIH, USA). All experiments were performed in triplicate, and statistical analysis was conducted using GraphPad Prism 9.0.

### 4.13. Immunofluorescence (IF) Assay

CAL27 cells grown on glass coverslips in 24-well plates were treated with varying concentrations of III-1a (0, 2.5, 5, or 10 μM) for 24 h. Cells were then fixed with 4% paraformaldehyde for 15 min at room temperature. Following three PBS washes, cells were permeabilized with 0.2% Triton X-100 for 10 min and blocked with 5% bovine serum albumin (BSA) for 1 h at room temperature. The samples were incubated overnight at 4 °C with anti-phospho-STAT3 primary antibody (AF3295; 1:100 dilution). After washing with PBS, cells were incubated with FITC-conjugated secondary antibody for 1 h at room temperature protected from light. Nuclei were counterstained with DAPI (Beyotime Biotechnology, Shanghai, China) for 5 min, followed by three washes with PBS containing 0.1% Tween-20 (PBST). Fluorescence images were acquired using a Leica SP8 confocal microscope system, with consistent acquisition parameters maintained across all samples.

### 4.14. Cell Transfection Experiment

CAL27 cells were inoculated into a 6-well plate with a density of 2 × 105 cells/well; 2 µg shSTAT3 plasmid DNA and 10 µL Lipofectamine-RNAiMAX transfection were added to 150 µL opti-MEM and gently mixed well. After standing at room temperature for 5 min, they were mixed well and left to rest at room temperature for 20 min. Finally, the mixture was dropped evenly into the 6-well plate. The cells were cultured with DMEM containing 1 µg/mL concentration of purinomycin after 48 h. The cells were screened for about 5 days. The surviving cells were inoculated into the 96-well plate with a density of 1 cells/well. Then the mimiclonal cells were continuously expanded to obtain stably transfected cell lines.

### 4.15. Small-Molecule Affinity Pull Down Assay for Target Protein Capture

CNBr-activated Sepharose 4B (CS4Bs) beads were swelled and activated with HCl solution and then equally aliquoted into two microcentrifuge tubes. Compound III-1a or an equal volume of DMSO (vehicle control) was added to each tube, followed by incubation with end-over-end rotation at 4 °C overnight to conjugate the small-molecule ligand to the beads. After blocking the remaining reactive sites on the beads with 0.1 M Tris-HCl buffer, equal volumes of cell lysate were added to both groups. The lysis buffer was composed of 50 mM Tris-HCl (pH 7.5), 5 mM EDTA, 150 mM NaCl, 1 mM DTT, 0.01% NP-40, 0.2 mM PMSF, and 1× protease inhibitor cocktail. The mixtures were incubated with gentle end-over-end rotation at 4 °C overnight to enable specific binding of target proteins to the immobilized small molecules. The bead pellets were harvested by centrifugation. The total concentration of proteins bound to the beads was quantified using the BCA protein assay, and the captured STAT3 proteins were further detected and qualitatively analyzed by Western blot.

### 4.16. Molecular Docking

The crystal structure of STAT3 (PDB ID: 6NUQ) was retrieved from the RCSB Protein Data Bank. The 2D chemical structure of compound was constructed in ChemBioDraw Ultra 14.0 and then converted into an energy-minimized 3D conformation using ChemBio3D. Molecular docking simulations were performed using AutoDock Tools (version 1.5.6), with the local search algorithm adopted for docking calculations. AutoDock software (version 1.5.6) outputs 10 top-ranked conformations, and the docking pose with the lowest binding energy was selected as the optimal conformation. The resulting structure was imported into PyMOL (version1.8) for structural visualization and figure generation, and the key interaction sites as well as the active binding pocket between compound III-1a and STAT3 were further characterized and analyzed.

### 4.17. MM-GBSA

The crystal structure of STAT3 (PDB ID: 6NUQ) was retrieved from the RCSB Protein Data Bank. The STAT3 structure was first processed by removing all water molecules and HETATM records. Hydrogen atoms were added and Gasteiger charges were assigned using AutoDockTools/MGLTools (version 1.2.7), followed by conversion into PDBQT format. The three-dimensional conformation of compound III-1a was likewise converted to PDBQT format. Molecular docking was performed using AutoDock Vina (version 1.2.7). The grid box was centered on the NZ atom of residue Lys244, with center coordinates of (−0.339, 11.239, 26.640) and dimensions of 24 × 24 × 24 Å. Docking parameters were set as follows: num_modes = 20, exhaustiveness = 48, energy range = 3 kcal/mol, and cpu = 48. The top 10 docking poses were retained, among which the top 5 poses were subjected to single-frame endpoint MM-GBSA rescoring using MMPBSA.py (version 14.0) within AmberTools (version 24.8). The ligand was parameterized with GAFF2 atom types and AM1-BCC charges.

### 4.18. Statistical Analysis

All experiments were performed in triplicate, with results presented as mean ± standard deviation (SD). Statistical analyses were conducted using GraphPad Prism 9.0 (GraphPad Software, San Diego, CA, USA). Differences between experimental groups and controls were assessed using two-tailed Student’s *t*-tests. For comparisons among three or more groups, one-way analysis of variance (ANOVA) was performed first, followed by an appropriate post hoc test for pairwise comparisons. Statistical significance was defined as *p* < 0.05, with the following significance levels indicated: * *p* < 0.05, ** *p* < 0.01, *** *p* < 0.001, and **** *p* < 0.0001.

## 5. Conclusions

Our study has identified a novel derivative of PL, designated as III-1a, which exhibits superior inhibitory activity against STAT3 compared to PL. III-1a markedly inhibits the activation of the JAK/STAT3 signaling pathway in OSCC cells and further suppresses the STAT3-mediated EMT process and induces ferroptosis, which consequently inhibits the invasion, migration, and proliferation of OSCC. However, the inhibitory effect of III-1a is reduced upon interference with STAT3. Thus, III-1a, as an inhibitor of the JAK/STAT3 pathway, demonstrates significant potential to be developed as a therapeutic agent for OSCC.

## Figures and Tables

**Table 1:** The inhibition rate (%) of PL and its derivatives on STAT3 activation.

Drugs	STAT3 Inhibition Rate (%)	Drugs	STAT3 Inhibition Rate (%)
I-1a	116.6 ± 0.3372	II-1c	118.06 ± 0.0957
I-1b	127.33 ± 0.6011	II-1d	132.68 ± 0.7502
I-1c	131.49 ± 0.1821	II-1e	119.46 ± 0.5475
I-1f	87.99 ± 0.6626	II-1K	113.70 ± 0.8545
I-187	102.35 ± 0.1020	III-1a	99.10 ± 0.9299
II-1a	133.03 ± 0.5125	PL	93.34 ± 0.1503
II-1b	129.09 ± 0.7101		

STAT3 inhibition rate (%) = (RLU_(IL6)_ − RLU_(compound)_)/(RLU_(IL6)_ − RLU_(control)_) × 100%.

**Table 2:** The IC_50_ of compound III-1a in 11 cancer cells.

Cell Lines	IC_50_ ± SD (μM)
CAL27	30 ± 2.13
A549	36.76 ± 0.87
SKOV3	44.72 ± 1.62
MCF-7	70.74 ± 10.10
PANC-1	149.2 ± 5.26
5637	32.76 ± 2.11
H460	72.71 ± 2.36
NOZ	64.61 ± 8.79
KB	55.05 ± 8.37
Kyse	88.7 ± 2.17
FADU	98.16 ± 4.36

## Data Availability

The data presented in this study are available in the article or in the Supplementary Materials.

## Supplementary Materials

The following supporting information can be downloaded at: https://www.mdpi.com/article/10.3390/molecules31142419/s1, Figure S1: (A) Heat map of the inhibition rate of STAT3 activation for PL and 23 derivatives at a concentration of 20 μM. (B) The IC50 Value of I-1f in HEK-293-STAT3. (C) The IC50 Value of III-1a in CAL27. (D) The IC50 Value of III-1a in A549. (E) The IC50 Value of III-1a in SKOV3. (F) The IC50 Value of III-1a in MCF-7. (G) The IC50 Value of III-1a in PANC-1. (H) The IC50 Value of III-1a in 5637. (I) The IC50 Value of III-1a in H460. (J) The IC50 Value of III-1a in NOZ. (K) The IC50 Value of III-1a in KB. (L) The IC50 Value of III-1a in Kyse. (M) The IC50 Value of III-1a in FADU; Figure S2: (A) Molecular docking analysis of I-1a and STAT3. (B) Molecular docking analysis of I-1b and STAT3. (C) Molecular docking analysis of I-1e and STAT3. (D) Molecular docking analysis of I-1f and STAT3. (E) Molecular docking analysis of I-187 and STAT3. (F) Molecular docking analysis of II-1a and STAT3. (G) Molecular docking analysis of II-1b and STAT3. (H) Molecular docking analysis of II-1c and STAT3. (I) Molecular docking analysis of II-1d and STAT3. (J) Molecular docking analysis of II-1e and STAT3. (K) Molecular docking analysis of II-1k and STAT3. (L) Molecular docking analysis of PL and STAT3; Table S1: Docking poses of III-1a and STAT3 by AutoDock Vina; Table S2: Docking of TOP 5 poses by endpoint MM-GBSA; Figure S3: Binding site of pose 2 in the III-1a-STAT3 complex from MM-GBSA analysis; Figure S4: (A) CNBr-activated sepharose 4B was coupled with III-1a (DMSO as control) and then incubated with CAL27 cell proteins. Western blotting was used to analyze the expression of STAT3 protein. (B) After CAL27 cells were treated with different concentrations of III-1a for 24 h, the ratio of live cells to total cells was analyzed. (C) MTT assay was used to analyze the cell viability after different concentrations of III-1a were treated with Erastin (20 μM) and Ferrostatin (5 μM) for 24 h. (D) MTT assay was used to analyze the cell viability after different concentrations of III-1a were treated with RSL3 (4 μM) and Ferrostatin (5 μM) for 24 h. * *p* < 0.05, ** *p* < 0.01; Figure S5: PL suppress tumor growth of oral squamous cell carcinoma. (A) Picture of nude mouse tumor with CAL27 cells. (B) Tumor weight of mice on the 21th day after administration. (C) Tumor volume in mice during the 21-day administration cycle. (D) The body weight of mice during the 21-day administration cycle.
