# Supplementary material for: A Derivative of Plumbagin Targets the JAK2/STAT3 Pathway to Inhibit the Progression of Oral Squamous Cell Carcinoma
Source: Molecules. 2026 Jul 9;31(14):2419. doi: 10.3390/molecules31142419 (PMC13413843; doi:10.3390/molecules31142419)
Supplement: Supplementary file 1 [file molecules-31-02419-s001.zip › molecules-4367695-supplementary.pdf]

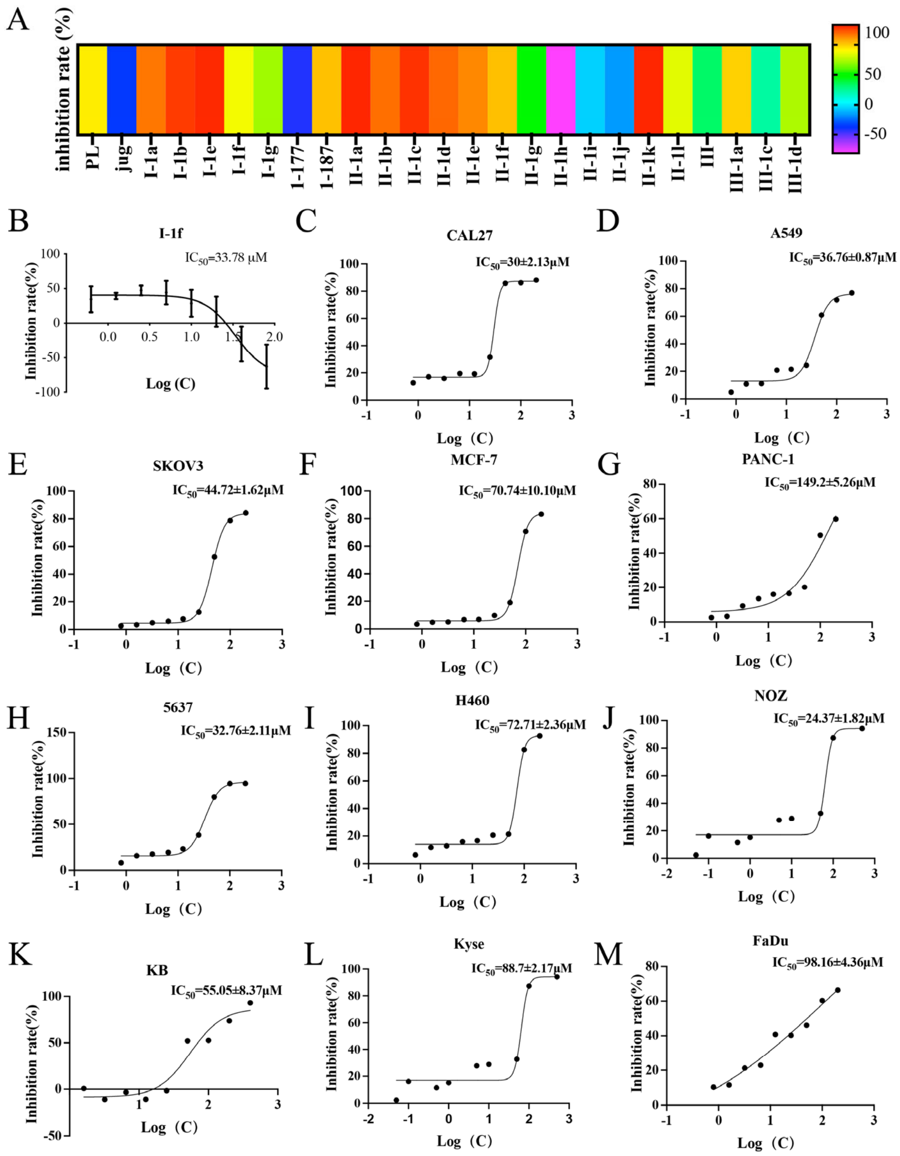

Figure S1. (A) Heat map of the inhibition rate of STAT3 activation for PL and 23 derivatives at a concentration of 20 μM. (B) The IC_{50} Value of I-1f in HEK-293-STAT3. (C) The IC_{50} Value of III-1a in CAL27. (D) The IC_{50} Value of III-1a in A549. (E) The IC_{50} Value of III-1a in SKOV3. (F) The IC_{50} Value of III-1a in MCF-7. (G) The IC_{50} Value of III-1a in PANC-1. (H) The IC_{50} Value of III-1a in 5637. (I) The IC_{50} Value of III-1a in H460. (J) The IC_{50} Value of III-1a in NOZ. (K) The IC_{50} Value of III-1a in KB. (L) The IC_{50} Value of III-1a in Kyse. (M) The IC_{50} Value of III-1a in FADU.

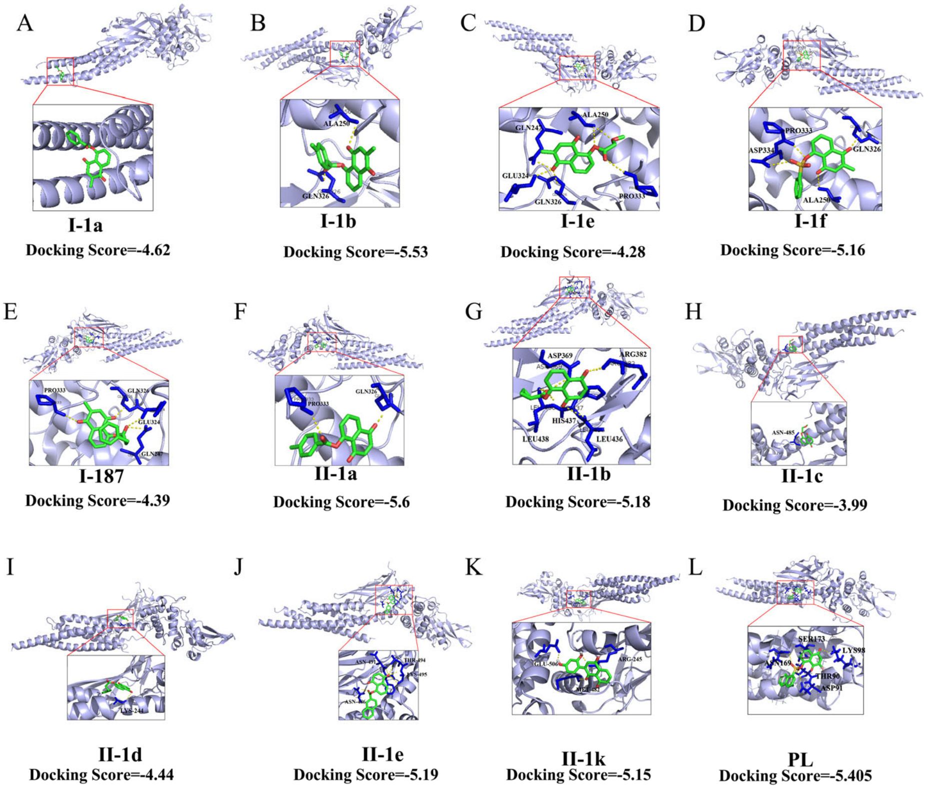

Figure S2. (A) Molecular docking analysis of I-1a and STAT3. (B) Molecular docking analysis of I-1b and STAT3. (C) Molecular docking analysis of I-1e and STAT3. (D) Molecular docking analysis of I-1f and STAT3. (E) Molecular docking analysis of I-187 and STAT3. (F) Molecular docking analysis of II-1a and STAT3. (G) Molecular docking analysis of II-1b and STAT3. (H) Molecular docking analysis of II-1c and STAT3. (I) Molecular docking analysis of II-1d and STAT3. (J) Molecular docking analysis of II-1e and STAT3. (K) Molecular docking analysis of II-1k and STAT3. (L) Molecular docking analysis of PL and STAT3.

Table S1. Docking poses of III-1a and STAT3 by AutoDock Vina.

Pose	Vina score	Top 5	Lys244 criterion	Shortest distance to Lys244 (Å)
1	-7.22	YES	Contact within 4A	3.6
2	-7.203	YES	H-bond possible	3.34
3	-7.188	YES	Contact within 4A	3.19
4	-7.169	YES	H-bond possible	3.27
5	-7.106	YES	H-bond possible	3.09
6	-7.068	NO	Contact within 4A	3.30
7	-7.045	NO	H-bond possible	2.80
8	-7.028	NO	Contact within 4A	3.27
9	-7.005	NO	H-bond possible	2.80
10	-6.993	NO	Contact within 4A	3.41

Table S2. Docking of TOP 5 poses by endpoint MM-GBSA.

Pose	Docking	ΔG bind	vdW	Elec.	Polar solv.	Nonpolar	recommend
1	-7.22	10.0466	-19.0363	-6.1301	39.4797	-4.2667	NO
2	-7.203	-2.2531	-36.1468	-16.5581	54.9893	-4.5375	YES
3	-7.188	1.0941	-20.9692	-8.1732	34.2776	-4.0411	NO
4	-7.169	2.6131	-30.8827	-34.1711	72.1331	-4.4662	NO

5	-7.106	4.4757	-18.9629	-12.018	39.4778	-4.0212	NO

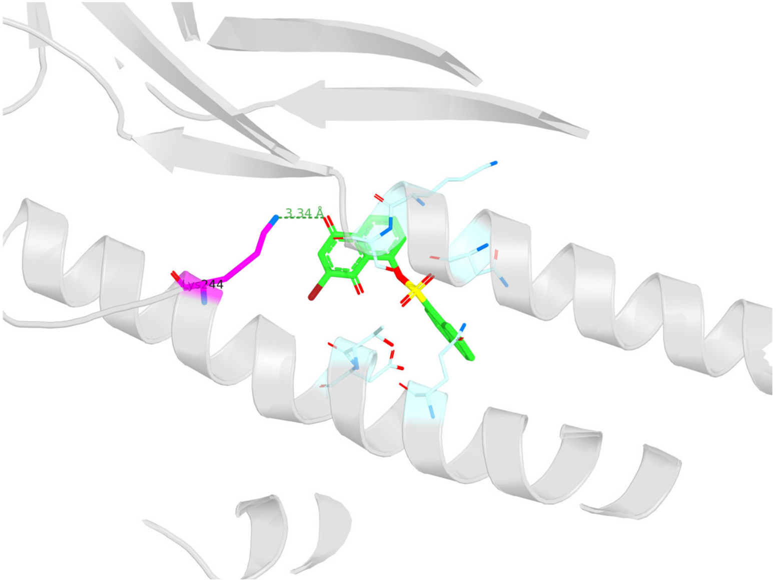

Figure S3. Binding site of pose 2 in the III-1a-STAT3 complex from MM-GBSA analysis.

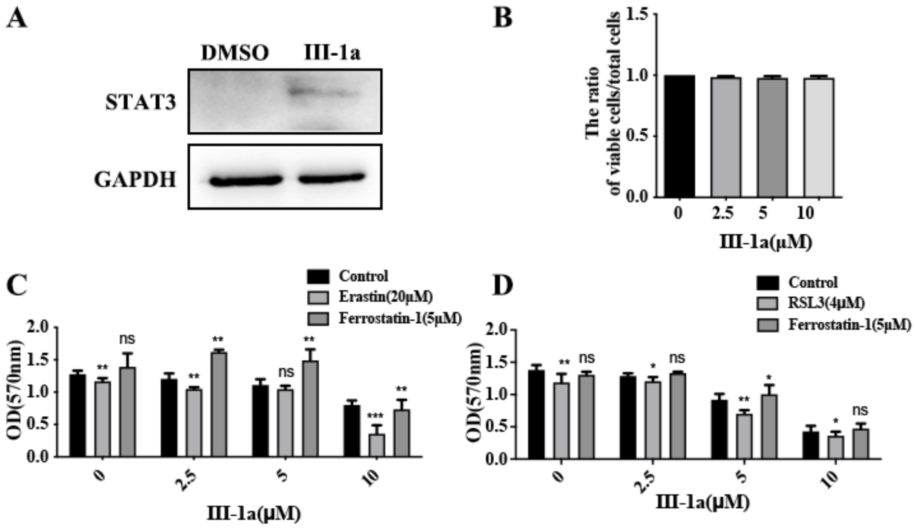

Figure S4. (A) CNBr-activated sepharose 4B was coupled with III-1a (DMSO as control) and then incubated with CAL27 cell proteins. Western blotting was used to analyze the expression of STAT3 protein. (B) After CAL27 cells were treated with different concentrations of III-1a for 24 hours, the ratio of live cells to total cells was analyzed. (C) MTT assay was used to analyze the cell viability after different concentrations of III-1a were treated with Erastin (20 μM) and Ferrostatin (5 μM) for 24 hours. (D) MTT assay was used to analyze the cell viability after different concentrations of III-1a were treated with RSL3 (4 μM) and Ferrostatin (5 μM) for 24 hours. *$p < 0.05$, **$p < 0.01$.

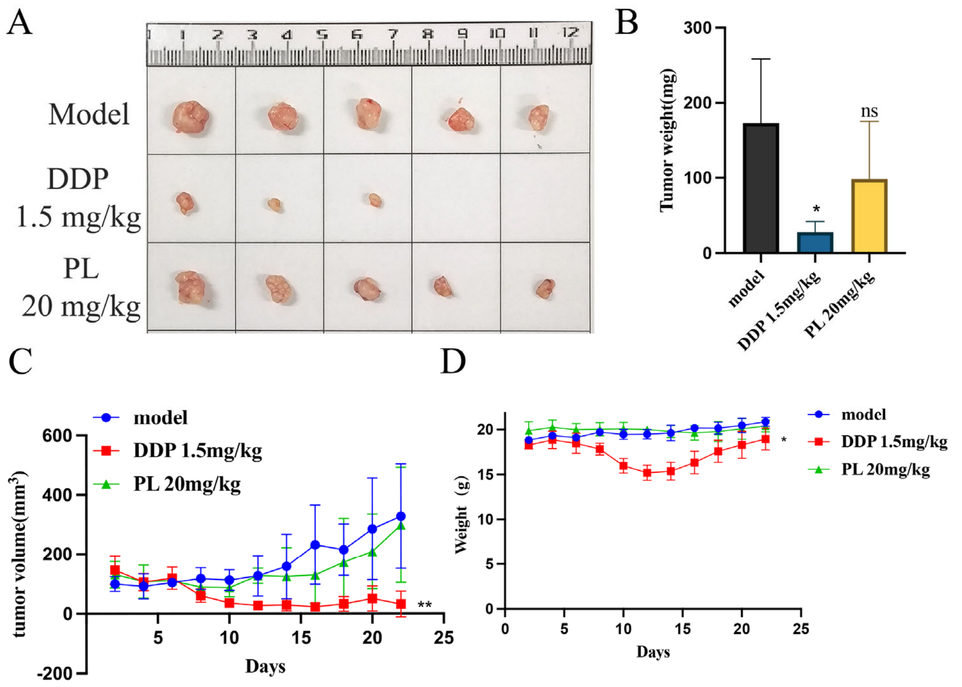

Figure S5. PL suppress tumor growth of oral squamous cell carcinoma. (A) Picture of nude mouse tumor with CAL27 cells. (B) Tumor weight of mice on the 21th day after administration. (C) Tumor volume in mice during the 21-day administration cycle. (D) The body weight of mice during the 21-day administration cycle.
